# An Updated Economic Assessment of Moxidectin Treatment Strategies for Onchocerciasis Elimination

**DOI:** 10.1093/cid/ciae054

**Published:** 2024-04-25

**Authors:** Hugo C Turner, Klodeta Kura, Barbara Roth, Annette C Kuesel, Sally Kinrade, Maria-Gloria Basáñez

**Affiliations:** UK Medical Research Council Centre for Global Infectious Disease Analysis, Department of Infectious Disease Epidemiology, School of Public Health, Imperial College London, London, United Kingdom; London Centre for Neglected Tropical Disease Research, Department of Infectious Disease Epidemiology, School of Public Health, Imperial College London, London, United Kingdom; UK Medical Research Council Centre for Global Infectious Disease Analysis, Department of Infectious Disease Epidemiology, School of Public Health, Imperial College London, London, United Kingdom; London Centre for Neglected Tropical Disease Research, Department of Infectious Disease Epidemiology, School of Public Health, Imperial College London, London, United Kingdom; Medicines Development for Global Health, Melbourne, Victoria, Australia; UNICEF/United Nations Development Progamme/World Bank/World Health Organization Special Programme for Research and Training in Tropical Diseases, World Health Organization, Geneva, Switzerland (retired); Medicines Development for Global Health, Melbourne, Victoria, Australia; UK Medical Research Council Centre for Global Infectious Disease Analysis, Department of Infectious Disease Epidemiology, School of Public Health, Imperial College London, London, United Kingdom; London Centre for Neglected Tropical Disease Research, Department of Infectious Disease Epidemiology, School of Public Health, Imperial College London, London, United Kingdom

**Keywords:** onchocerciasis, elimination, ivermectin, moxidectin, economic assessment

## Abstract

**Background:**

Concerns that annual mass administration of ivermectin, the predominant strategy for onchocerciasis control and elimination, may not lead to elimination of parasite transmission (EoT) in all endemic areas have increased interest in alternative treatment strategies. One such strategy is moxidectin. We performed an updated economic assessment of moxidectin- relative to ivermectin-based strategies.

**Methods:**

We investigated annual and biannual community-directed treatment with ivermectin (aCDTI, bCDTI) and moxidectin (aCDTM, bCDTM) with minimal or enhanced coverage (65% or 80% of total population taking the drug, respectively) in intervention-naive areas with 30%, 50%, or 70% microfilarial baseline prevalence (representative of hypo-, meso-, and hyperendemic areas). We compared programmatic delivery costs for the number of treatments achieving 90% probability of EoT (EoT_90_), calculated with the individual-based stochastic transmission model EPIONCHO-IBM. We used the costs for 40 years of program delivery when EoT_90_ was not reached earlier. The delivery costs do not include drug costs.

**Results:**

aCDTM and bCDTM achieved EoT_90_ with lower programmatic delivery costs than aCDTI with 1 exception: aCDTM with minimal coverage did not achieve EoT_90_ in hyperendemic areas within 40 years. With minimal coverage, bCDTI delivery costs as much or more than aCDTM and bCDTM. With enhanced coverage, programmatic delivery costs for aCDTM and bCDTM were lower than for aCDTI and bCDTI.

**Conclusions:**

Moxidectin-based strategies could accelerate progress toward EoT and reduce programmatic delivery costs compared with ivermectin-based strategies. The costs of moxidectin to national programs are needed to quantify whether delivery cost reductions will translate into overall program cost reduction.

## Teaser

Moxidectin-based strategies could accelerate progress toward the elimination of onchocerciasis transmission and reduce programmatic delivery costs compared with ivermectin-based strategies. These results are particularly relevant for highly endemic areas where biannual moxidectin treatment will likely be necessary to achieve elimination.

Onchocerciasis is a parasitic disease caused by the filarial nematode *Onchocerca volvulus* and transmitted among humans through the bites of several *Simulium* blackfly species [1]. The World Health Organization (WHO) and endemic countries target elimination of parasite transmission (EoT) [2]. To date, EoT has been verified by WHO in the 8 foci in 4 of the 6 countries in the Americas (Colombia, Ecuador, Guatemala, and Mexico). In the Americas, the population at risk was less than 0.6 million [3, 4]. In contrast, in sub-Saharan Africa, 244 million people are currently estimated to live in areas where interventions to eliminate transmission are required [5].

Currently, the cornerstone of onchocerciasis control and elimination strategies is annual mass drug administration (MDA) with ivermectin (Mectizan) using community-directed treatment with ivermectin (CDTI) [6]. Mectizan is donated by Merck & Co Inc, which in 1987 committed to supplying it “as much as necessary for as long as necessary” to onchocerciasis-endemic countries [7, 8]. Concerns that annual CDTI (aCDTI) may not achieve EoT in all endemic foci [2] have turned attention to evaluating alternative treatment strategies [9–11], including increased CDTI frequency or the use of alternative drugs. One alternative is moxidectin. The phase 3 clinical trial found that a single dose of moxidectin achieves lower skin microfilarial loads in more people and for longer than a single ivermectin dose [12, 13], reducing the potential for transmission between treatment rounds and increasing the feasibility of achieving EoT. In 2018, the US Food Drug Administration (FDA) approved moxidectin for treatment of onchocerciasis in those aged ≥12 years [14–16].

A previous modeling study concluded that annual moxidectin and biannual ivermectin MDA would achieve similar reductions in program duration relative to annual ivermectin treatment [17]. Unlike biannual CDTI (bCDTI), annual community-directed treatment with moxidectin (aCDTM) would not incur an increase in delivery costs and, therefore, would reduce the projected total long-term programmatic cost (assuming moxidectin is donated to endemic countries). Turner et al [17] used a previous version of the population-based, deterministic EPIONCHO epidemiological model and evaluated the number of treatment rounds and costs needed to achieve the microfilarial prevalence-based component of the provisional Operational Thresholds for Treatment Interruption followed by Surveillance, proposed by the African Programme for Onchocerciasis Control in 2010 [17, 18]. Therefore, Turner et al [17] did not investigate the probabilities of reaching EoT with different MDA strategies.

This study provides an updated economic assessment of moxidectin- relative to ivermectin-based MDA strategies including biannual CDTM (bCDTM), which was not included in the previous study [17]. We compare the projected programmatic delivery costs of different treatment strategies using the number of treatment rounds needed to achieve EoT under the epidemiological scenarios recently investigated by Kura et al [19] with the individual-based stochastic onchocerciasis (EPIONCHO-IBM) transmission model [20].

## METHODS

### Model and Scenarios Investigated

This analysis is based on the EPIONCHO-IBM epidemiological projections presented by Kura et al [19], who investigated the probability of achieving EoT for different durations of aCDTI, bCDTI, aCDTM, and bCDTM for a range of epidemiological scenarios. We calculated the number of treatment rounds necessary to achieve a 90% probability of EoT (referred to here as EoT_90_; see Supplementary Material for details on the calculation of EoT_90_) for hypo-, meso-, and hyperendemic areas (represented by 30%, 50%, and 70% baseline microfilarial prevalence, respectively) without prior intervention history. For biannual treatment strategies, the projected number of years of treatment required to reach EoT_90_ was rounded up to a whole number.

EPIONCHO-IBM is an individual host-based stochastic onchocerciasis transmission model [20] that models drug effects in terms of temporal dynamics of the “microfilaricidal effect” (killing of microfilariae) and “embryostatic effect” (temporary disruption of microfilarial production by adult female worms) of both ivermectin and moxidectin (Supplementary Figure 1). The model also includes a “permanent sterilizing effect” on adult (macrofilaria) female worms (ie, a cumulative, irreversible reduction of female worm microfilarial productivity). This reduction is assumed to be 35% per standard (150 µg/kg) dose of ivermectin [21]. Since no data yet exist on the effect of multiple doses of 8 mg moxidectin (the dose approved by the FDA), we assumed the same permanent sterilizing effect as for ivermectin (see Supplementary Table 1 for parameterization of drug effects). This was varied in the sensitivity analysis (Table 1) reflective of potential differences between the permanent sterilizing effect of ivermectin and moxidectin due to the latter's longer half-life and duration of exposure to the drug after dosing compared with ivermectin [24].

**Table 1.** Overview of Treatment Strategies and Scenarios Investigated

Treatment Strategies, Epidemiological and Programmatic Scenarios, Drug Effect Assumptions, and Cost Considerations	
Treatment strategies	4 MDA strategies were investigated: aCDTI: annual community-directed treatment with ivermectin bCDTI: biannual (6-monthly) community-directed treatment with ivermectin aCDTM: annual community-directed treatment with moxidectin bCDTM: biannual (6-monthly) community-directed treatment with moxidectin It was also assumed that, as with ivermectin, individuals aged ≥5 years would be eligible for moxidectin treatment (as in [19]).
Baseline prevalence (endemicity) levels	3 baseline prevalence (endemicity) settings were considered: Hypoendemic: 30% microfilarial prevalence Mesoendemic: 50% microfilarial prevalence Hyperendemic: 70% microfilarial prevalence All of the above are calculated over all individuals aged ≥5 years in the modeled population (of 440 individuals). To allow a clear comparison of scenarios, it was assumed that the settings had not undergone any previous treatment (ie, they were treatment-naive).
Coverage scenarios^a,b^	2 coverage scenarios were modeled: Minimal: 65% therapeutic coverage *of the total population* and 5% systematic nonadherence Enhanced: 80% therapeutic coverage *of total population* and 1% systematic nonadherence
Drug effects	EPIONCHO-IBM incorporates the following 3 drug effects: Microfilaricidal effect: killing of microfilariae Embryostatic effect: temporary disruption of microfilarial production by adult female worms Permanent sterilizing effect: cumulative, irreversible reduction of female worm microfilarial productivity The first 2 effects are modeled so as to capture their temporal dynamics following treatment with either ivermectin or moxidectin (Supplementary Figure 1). The third effect is modeled as a fixed reduction in microfilarial production, per treatment round, assumed to be 35% per standard dose of ivermectin (150 µg/kg) and moxidectin (8 mg). In the sensitivity analysis, we consider a scenario in which ivermectin had half the magnitude of the permanent sterilizing effect compared with moxidectin (with ivermectin causing an irreversible reduction in microfilarial production of 17.5% per treatment round and moxidectin causing 35% reduction per treatment round).
Programmatic delivery costs	The annual economic cost of the aCDTI and aCDTM strategies was assumed to be $50 535 per 100 000 individuals. 2 assumptions were explored regarding the increase in total delivery cost per year when increasing treatment frequency from annual to biannual: Biannual treatment would increase the total delivery cost per year by 60% compared with annual treatment [22]. Biannual treatment would increase the total delivery cost per year by 100% compared with annual treatment.

Minimal and enhanced coverage correspond to ≍80% and ≍100% of the eligible population taking the drug. Individuals aged <5 years and pregnant women (as well as those breastfeeding a baby younger than 1 week) are not part of the eligible population.

Abbreviations: aCDTI, annual community-directed treatment with ivermectin; aCDTM; annual community-directed treatment with moxidectin; bCDTI, biannual community-directed treatment with ivermectin; bCDTM, biannual community-directed treatment with moxidectin.

^a^Coverage is the proportion of the total population receiving ivermectin or moxidectin at each treatment round; systematic nonadherence is the proportion of the eligible population who never receive treatment.

^b^These coverage levels (of total population) were chosen because a minimum of 65% has been indicated for onchocerciasis control as a public health problem, while reaching 80% coverage has been proposed as the benchmark for elimination of transmission [23].

Table 1 summarizes the treatment strategies, epidemiological and programmatic scenarios, drug effects assumptions, and cost considerations for our analysis.

### Programmatic Delivery Costs

We projected the total programmatic delivery costs for the treatment strategies investigated to achieve EoT_90_ or for a maximum of 40 years. The costs within this analysis were considered from the healthcare provider’s perspective and did not include the economic value of the drugs.

The annual economic cost of the aCDTI strategy was assumed to be $50 535 per 100 000 individuals. This was based on costs collected in Ghana [22], adjusting for inflation to 2021 prices (using gross domestic product deflators related to Ghana [25] and accounting for changes in the exchange rate [26]). The cost of aCDTM was assumed to be the same as that of aCDTI.

Two scenarios were considered regarding the increase in costs with a biannual treatment strategy. The former was that biannual treatment would increase the total delivery cost per year by 60% compared with annual treatment (based on [22]). The latter was that the total cost per year for delivering MDA biannually would be double (a 100% increase) the cost for annual MDA. It was assumed that the annual delivery costs were the same for the different coverage scenarios (Table 1) [27].

### Economic Analysis

The projected total programmatic delivery cost to EoT_90_ with bCDTI, aCDTM, and bCDTM strategies was calculated relative to the costs with aCDTI (ie, the standard strategy of annual treatment with ivermectin was the comparator). A 40-year time horizon was considered within the analysis (ie, the costs for any scenario not reaching EoT_90_ within 40 years were those incurred over 40 years within the cost calculations). The costs are in 2021 US dollars and were discounted at 3% per year (following WHO guidelines [28]).

We adhered to the 5 principles of the Neglected Tropical Disease (NTD) Modelling Consortium regarding policy-relevant items for reporting models in epidemiology of NTDs for good practice in NTD modeling [29] (Supplementary Table 2).

## RESULTS

### Projected Program Duration

**Figure 1.**
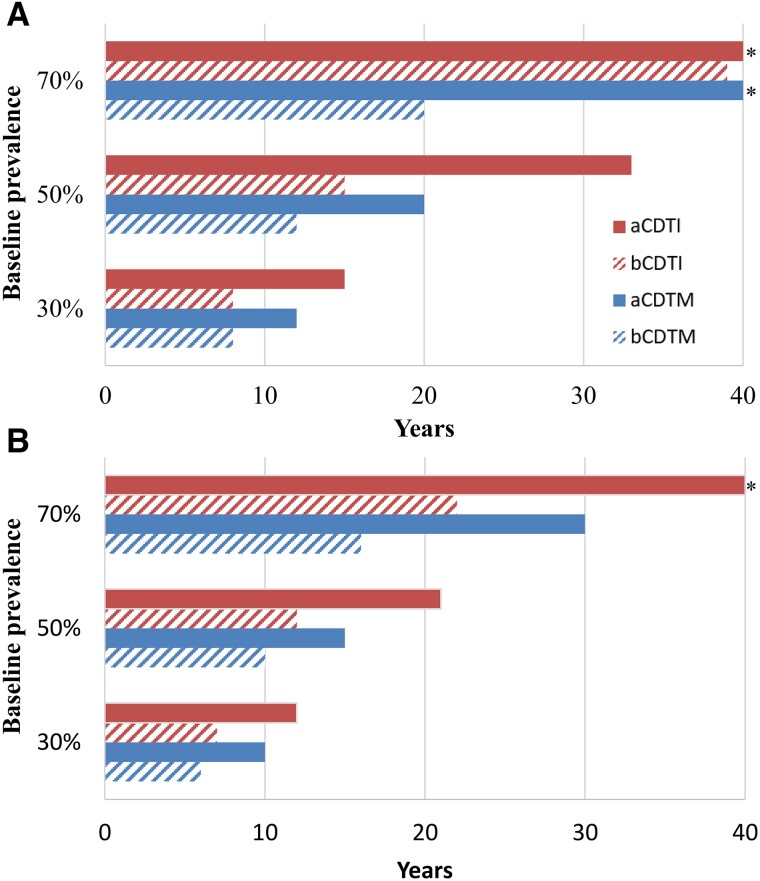
Projected number of years to achieve 90% probability of elimination of transmission (EoT_90_) for 3 endemicity levels (baseline microfilarial prevalence) under 2 treatment coverage scenarios. *A*, Minimal coverage: 65% therapeutic coverage of total population and 5% systematic nonadherence. *B*, Enhanced coverage: 80% therapeutic coverage of total population and 1% systematic nonadherence. Simulations assume that ivermectin and moxidectin exert the same magnitude of the permanent sterilizing effect, that is, both drugs cause a cumulative, permanent reduction of microfilarial production of 35% per treatment round with a standard dose (150 µg/kg for ivermectin and 8 mg for moxidectin). * Elimination not attained within the 40-year time horizon. See Supplementary Material for definition and calculation of the probability of EoT. Abbreviations: aCDTI, annual community-directed treatment with ivermectin; aCDTM; annual community-directed treatment with moxidectin; bCDTI, biannual community-directed treatment with ivermectin; bCDTM, biannual community-directed treatment with moxidectin.

The projected program durations needed to achieve EoT_90_ for the different treatment strategies are shown in Figure 1. In terms of the absolute reduction in the number of years of treatment required relative to aCDTI, the benefits of moxidectin-based strategies were greater in settings with a higher baseline endemicity. The relative gain for moxidectin- versus ivermectin-based strategies was slightly smaller under the enhanced coverage scenario (Figure 1*B* ) compared with the minimal coverage scenario (Figure 1*A* ).

When assuming that both drugs exert the same magnitude of the permanent sterilizing effect, aCDTM did not reduce program duration to EoT_90_ relative to aCDTI to the same degree as bCDTI. bCDTM consistently achieved the greatest reduction in program duration (Figure 1).

In settings with 70% baseline microfilarial prevalence, EoT_90_ was projected to not be feasible with aCDTI, even with enhanced coverage (Figure 1*B* ). In such settings, a biannual treatment strategy was projected to be needed to achieve EoT_90_ (in less than 25 years for bCDTI at enhanced coverage), with bCDTM being the most effective, reaching EoT_90_ in less than 20 years, both with minimal and enhanced coverage.

Supplementary Table 3 shows the projected number of treatments per 100 000 individuals needed for the different scenarios to achieve EoT_90_ or the number of treatments delivered over 40 years for strategies not achieving EoT_90_. This illustrates that even modest reductions in program duration can have substantial programmatic implications on a large scale.

### Projected Programmatic Delivery Costs

Table 2 shows the projected duration (in years) and the relative (to aCDTI) programmatic delivery costs (in percent) to achieve EoT_90_ with aCDTI, bCDTI, aCDTM, and bCDTM within the 40-year time horizon investigated, assuming that the total cost per year would increase by 60% (Table 2*A* ) or by 100% (Table 2*B* ) when treating biannually.

**Table 2.** Projected Number of Years to Achieve 90% Probability of Elimination of Transmission and Relative Programmatic Delivery Cost of the Different Treatment Strategies Compared With Annual Community-Directed Treatment With Ivermectin Over the 40-Year Time Horizon

	Minimal Coverage Scenario			Enhanced Coverage Scenario		
Strategy	Baseline Microfilarial Prevalence			Baseline Microfilarial Prevalence		
A. Treatment duration (in years) and relative cost (in percent) compared with annual community-directed treatment with ivermectin assuming that the total cost of treatment per year would increase by 60% when treating biannually						
	30%	50%	70%	30%	50%	70%
aCDTI	15 (0%)	33 (0%)	40+ (0%)	12 (0%)	21 (0%)	40+ (0%)
bCDTI	8 (−6%)	15 (−8%)	39 (+58%)^a^	7 (0%)	12 (+3%)	22 (+10%)^a^
aCDTM	12 (−17%)	20 (−28%)	40+ (0%)^a^	10 (−14%)	15 (−23%)	30 (−15%)^a^
bCDTM	8 (−6%)	12 (−23%)	20 (+3%)^a^	6 (−13%)	10 (−11%)	16 (−13%)

	Minimal coverage scenario			Enhanced coverage scenario		
Strategy	Baseline microfilarial prevalence			Baseline microfilarial prevalence		
B. Treatment duration (in years) and relative cost (in percent) compared with aCDTI assuming that the total cost of treatment per year would increase by 100% when treating biannually						
	30%	50%	70%	30%	50%	70%
aCDTI	15 (0%)	33 (0%)	40+ (0%)	12 (0%)	21 (0%)	40+ (0%)
bCDTI	8 (+18%)	15 (+15%)	39 (+97%)^a^	7 (+25%)	12 (+29%)	22 (+38%)^a^
aCDTM	12 (−17%)	20 (−28%)	40+ (0%)^a^	10 (−14%)	15 (−23%)	30 (−15%)^a^
bCDTM	8 (+18%)	12 (−4%)	20 (+29%)^a^	6 (+9%)	10 (+11%)	16 (+9%)^a^

The values in parentheses represent the projected relative proportions (in percent) of total programmatic delivery cost of the different treatment strategies compared with aCDTI over a 40-year time horizon and exclude the economic value of the drugs. Values of 0% signify that the costs are the same as for aCDTI; those accompanied by a minus sign indicate a relative reduction in cost; those accompanied by a plus sign indicate a relative increase in cost. See Table 1 for definitions of treatment strategies and coverage scenarios. It was assumed that both drugs exert a cumulative, permanent reduction of microfilarial production by adult female worms of 35% per treatment round with a standard dose of ivermectin (150 µg/kg) or moxidectin (8 mg). The corresponding projections of the total (absolute) programmatic delivery costs are presented in Supplementary Tables 4 and 5.

Abbreviations: aCDTI, annual community-directed treatment with ivermectin; aCDTM; annual community-directed treatment with moxidectin; bCDTI, biannual community-directed treatment with ivermectin; bCDTM, biannual community-directed treatment with moxidectin.

^a^Since elimination of transmission was not attained with aCDTI within the 40-year time horizon, the relative costs are calculated based on costs of 40 years of aCDTI.

Assuming a 60% increase and in areas with baseline microfilarial prevalence of 30% and 50% under minimal coverage, bCDTI was equally or more expensive than aCDTM and bCDTM. With enhanced coverage, the programmatic delivery costs for aCDTM and bCDTM were lower than for aCDTI and bCDTI for all endemicity levels. aCDTM had a similar or lower cost than bCDTM, and both were cheaper than bCDTI. The greatest reductions in program duration were achieved by bCDTM (Figure 1*B*, Table 2*A* ).

When assuming that the total cost per year for biannual MDA is twice that for annual MDA, the cost for bCDTI and bCDTM increased, influencing their relative cost to aCDTI (see Table 2*A* versus Table 2*B* ). Under this assumption, aCDTM was the cheapest strategy for all modeled scenarios (Supplementary Table 5). The relative cost of bCDTM compared with aCDTI ranged from a 4% reduction to a 29% increase under minimal coverage, with substantially lower increase (by 9% to 11%) under enhanced coverage. As shown previously [19], bCDTM was projected to generate the greatest reductions in program duration and could achieve EoT_90_ in hyperendemic settings, where it was not feasible with other strategies within the time horizon considered. The relative cost of bCDTM was highest for the 70% baseline microfilarial prevalence scenario under minimal coverage but not under enhanced coverage (Table *2B* ). Since EoT was not achieved with aCDTI within the 40-year time horizon for such settings, the relative costs, calculated based on costs of 40 years of aCDTI, underestimate potential cost savings.

### Assuming Different Magnitudes of the Permanent Sterilizing Effect on *O. volvulus* of Ivermectin and Moxidectin

A sensitivity analysis explored the influence on modeled outputs of a difference in the magnitude of the permanent sterilizing effect of the 2 drugs. When ivermectin's effect was assumed to be half that of moxidectin (17.5% versus 35% reduction in microfilarial production per treatment round [19]), aCDTM was projected to be broadly equivalent to bCDTI in terms of reduction in program duration to achieve EoT_90_ under minimal coverage (Supplementary Figure 2 and [19]).

Under these assumptions, the relative cost of bCDTI increased and, depending on the scenario, was similar to or higher than that of aCDTI (Supplementary Table 6). bCDTI was not projected to achieve EoT_90_ within 40 years in settings with 70% baseline microfilarial prevalence (Supplementary Figure 2). In these settings, compared with aCDTI, moxidectin-based strategies have an equivalent cost (aCDTM) or a somewhat greater cost (bCDTM), but bCDTM achieved EoT_90_ at around 20 years while this was not feasible with any other strategy. In areas with 30% or 50% baseline microfilarial prevalence, moxidectin-based strategies were cost-saving. The total costs of aCDTM and bCDTM were similar, but bCDTM resulted in the greatest reductions in program duration (Supplementary Table 7, Supplementary  Figure 2).

## DISCUSSION

As indicated by Kura et al, moxidectin-based strategies have the potential to accelerate progress toward onchocerciasis EoT relative to ivermectin-based strategies [19]. Our analyses suggest that moxidectin-based strategies can reduce programmatic delivery costs to achieve EoT_90_ relative to ivermectin-based strategies. While aCDTM did not reduce the number of years needed to achieve EoT_90_ compared with aCDTI as much as bCDTI, the lower number of treatments needed resulted in aCDTM programmatic delivery costs being lower than those for bCDTI. Furthermore, the projection that bCDTM is more effective than bCDTI in accelerating progress toward EoT_90_ translated into bCDTM delivery being cheaper than bCDTI.

The degree of programmatic delivery cost reduction relative to aCDTI varied across different epidemiological settings. The cost reduction with moxidectin- relative to ivermectin-based strategies is particularly relevant for highly endemic areas for which EPIONCHO-IBM suggests that bCDTM is needed to reach EoT_90_ within 20 years for both coverage scenarios (Figure 1 and [19]). Such areas have the potential to be very costly to NTD programs in the long term, and continuing transmission in these areas could conceivably reintroduce infection to others [30].

The results regarding the long time frame required to reach the projected EoT_90_ [19] highlight the need for stakeholders and donors in this area to have evidence-based goals and long-term time horizons. This also underscores the continued need for the development and implementation of alternative and/or complementary onchocerciasis interventions (eg, macrofilaricidal drugs and sustainable vector control).

In the context of the current elimination goals, the projected difference between the coverage/adherence scenarios highlights the importance of mobilizing health education/community engagement to ensure that coverage and adherence are as high as possible.

The projected magnitude of these cost reductions varied across different epidemiological settings and are sensitive to assumptions regarding delivery costs when increasing treatment frequency from yearly to every 6 months, as well as to assumptions regarding the permanent sterilizing effect of ivermectin compared with moxidectin, which remains uncertain [19].

An explanation of the differences between our results and those presented in the previous economic analysis [17] is provided in the Supplementary Material.

In this study, we evaluated the relative programmatic delivery costs of different treatment strategies compared with aCDTI for 3 scenario-based baseline endemicity settings (Table 1). It is, therefore, not focused on any particular country/endemic area and should be generally illustrative of moxidectin's potential in a range of African savannah settings. To provide proof of principle of the relative programmatic delivery costs for these different treatment strategies, our analysis assumed intervention-naive scenarios and closed populations. While some intervention-naive areas exist [31] (particularly, but not solely, hypoendemic areas not prioritized for CDTI when control of onchocerciasis as a public health problem was targeted), the majority of endemic areas have implemented aCDTI for years (and some have a history of other interventions, including vector control and bCDTI). Modeling indicates that when aCDTI has made little progress, switching to moxidectin-based strategies will reduce the number of years required to reach EoT_90_ [19, 32]. However, the delivery cost reductions will likely differ from those presented here. Future work should focus on country-specific settings, taking into account baseline infection prevalence, prevailing vector species and biting rates, previous interventions and coverage data, as well as country-specific costs for annual and, if available, biannual treatment delivery. To maximize usefulness for countries, other factors should be included, such as transmission seasonality, to guide optimal annual or biannual treatment timing [17]. Including spatial heterogeneity in endemicity (and vector biting rates) within transmission zones would help us to better understand the consequences of people and/or vector movement between communities on achieving and maintaining EoT [30], with potential repercussions on required treatment duration, number of treatments, and associated delivery costs to achieve EoT across the whole transmission zone. The fact that bCDTM is projected to reduce the difference in the number of treatments needed to achieve EoT across different endemicity settings (ie, less difference between number of treatment rounds needed to achieve EoT in areas ranging from lower to higher baseline prevalence compared with ivermectin-based strategies) could be an added advantage for country programs. It is important to consider that as MDA programs scale down, the cost per treatment will increase for the remaining areas in need of treatment [27], which will increase the programmatic cost for areas that lag behind.

Given the paucity of data for both moxidectin and ivermectin with which to parameterize the assumed permanent sterilizing effect of the drugs [17, 19], and considering moxidectin's longer half-life and superior clearance of skin microfilariae (Supplementary Figure 1 and [12, 13, 16, 24]), we investigated the influence of keeping the value of moxidectin's effect the same as for the results discussed above but halving that of ivermectin by way of sensitivity analysis. Under this assumption, epidemiological projections for aCDTM and bCDTI were closely aligned [19], and the relative (to aCDTI) cost reductions of moxidectin-based strategies increased. This suggests that a better understanding of the operation and magnitude of any permanent sterilizing effect on *O. volvulus* by both drugs is a pressing need to reduce this uncertainty.

The extent to which the projected delivery cost reduction for moxidectin-based strategies will lead to overall program cost reductions will depend on the cost of moxidectin to countries. As a not-for-profit organization, Medicines Development for Global Health, the sponsor of moxidectin, works in a different financial context than Merck & Co, Inc, which donates Mectizan through the Mectizan Donation Program. A sustainable model for financing the manufacture and supply of moxidectin is currently being sought. This includes potential funding by a donor or donors to remove (or at least minimize) costs to national programs. That said, if moxidectin is not donated, the potential additional cost will need to be taken into account when countries are considering its adoption.

Comprehensive data regarding the costs of MDA delivery and, in particular, the relative cost of biannual and annual treatment are lacking [33]. Our assumption of a 60% increase in the cost of biannual versus annual treatment is based on a single primary costing study in Ghana [23]. To address this limitation, we projected delivery costs for the worst-case scenario in which the annual cost of delivering biannual MDA is double that of annual treatment.

We did not include the additional costs incurred in the first couple of years of moxidectin-based strategies due to additional training of healthcare workers and community drug distributors on important differences between moxidectin and ivermectin. These will affect total programmatic delivery the less the longer the required program duration.

For comparability, we assumed that individuals aged ≥5 years are eligible for moxidectin as for ivermectin treatment. While the current FDA approval for moxidectin is only for those aged ≥12 years [14, 16], work is ongoing toward obtaining FDA approval for treatment of children aged 4‒11 years [34]. This will be an important step for moxidectin's usefulness to national programs, as the need to treat children aged 4‒11 years with ivermectin but children aged ≥12 years with moxidectin would increase the complexity of MDA implementation and represent a significant barrier to moxidectin use.

Moxidectin exerts a more potent initial microfilaricidal effect than ivermectin [35]. It is, therefore, likely that, as with ivermectin, moxidectin will be contraindicated in individuals with heavy *Loa loa* microfilaraemia due to the risk of severe and/or serious adverse reactions due to the microfilaricidal activity against *L. loa* [36]. Therefore, there is a need to identify alternative strategies in onchocerciasis–loiasis co-endemic settings and investigate moxidectin's potential role in test-and-not-treat strategies as for ivermectin [37]. The first study comparing the safety and efficacy of moxidectin and ivermectin in *L. loa*-infected individuals has completed safety follow-up [38].

This analysis focused on the years of treatment and programmatic delivery costs required to reach the projected EoT_90_. However, it remains important for future work to also investigate the projected health impacts of onchocerciasis interventions and the elimination of onchocerciasis as a public health problem. In this context, it is important to note that there are settings with ongoing morbidity despite long-term CDTI [39].

## CONCLUSIONS

Modeling suggests that moxidectin-based strategies could accelerate progress toward onchocerciasis EoT relative to aCDTI [19]. Our analysis projected that moxidectin could also reduce programmatic delivery costs. In terms of program duration and programmatic delivery costs, aCDTM could be an optimal strategy where only annual treatment is feasible, while bCDTM is preferable where biannual treatment can be implemented and the only strategy capable of achieving EoT_90_ in highly endemic areas. The extent to which programmatic delivery cost reductions will translate into overall programmatic cost reductions will depend on the cost of moxidectin for endemic countries.

## Supplementary Data

Supplementary materials are available at *Clinical Infectious Diseases* online. Consisting of data provided by the authors to benefit the reader, the posted materials are not copyedited and are the sole responsibility of the authors, so questions or comments should be addressed to the corresponding author.

## Supplementary Material

ciae054_Supplementary_Data

## Notes

***Author contributions.*** Conceptualization: H. C. T., K. K., B. R., A. C. K., S. K., M. G. B. Formal analysis: H. C. T., K. K., M. G. B. Funding acquisition: H. C. T., S. K., M. G. B. Investigation and methodology: H. C. T., K. K., M. G. B. Project administration: H. C. T., B. R., S. K., M. G. B. Resources: H. C. T., K. K., M. G. B. Software: K. K., M. G. B. Supervision: S. K., M. G. B. Visualization: H. C. T., M. G. B. Writing the original draft: H. C. T., S. K., M. G. B. Writing: review and editing: H. C. T., B. R., A. C. K., S. K., M. G. B.

***Acknowledgments.*** The authors thank Mark Sullivan, Jonathan Hamley, Martin Walker, and Philip Milton for their insights and contributions to the epidemiological simulation projections and the Neglected Tropical Disease (NTD) Modelling Consortium for helpful discussions. They also acknowledge those involved with conducting the phase 3 clinical trial of moxidectin: Nicholas O. Opoku, Didier K. Bakajika, Eric M. Kanza, Hayford Howard, Germain L. Mambandu, Amos Nyathirombo, Maurice M. Nigo, Kambale Kasonia, Safari L. Masembe, Mupenzi Mumbere, Kambale Kataliko, Jemmah P. Larbelee, Mawolo Kpawor, Kpehe M. Bolay, Fatorma Bolay, Sampson Asare, Simon K. Attah, George Olipoh, Michel Vaillant, and Christine M. Halleux.

***Supplement sponsorship.*** This article appears as part of the supplement “New Tools and Nuanced Interventions to Accelerate Achievement of 2030 Roadmap for Neglected Tropical Diseases,” sponsored by funding of Professor T. Déirdre Hollingsworth's research by the Li Ka Shing Foundation at the Big Data Institute, Li Ka Shing Centre for Health Information and Discovery, University of Oxford; and funding of the NTD Modelling Consortium by the Bill & Melinda Gates Foundation (INV-030046).

***Financial support.*** H. C. T. received funding from Medicines Development for Global Health to conduct this analysis as a consultant. H. C. T., K. K., and M. G. B. acknowledge funding from the UK Medical Research Council (MRC) Centre for Global Infectious Disease Analysis (MR/X020258/1), funded by the UK MRC. This UK-funded award is carried out in the frame of the Global Health EDCTP3 Joint Undertaking. K. K. and M. G. B. also acknowledge funding by the Bill & Melinda Gates Foundation through the NTD Modelling Consortium (grant INV-030046). For the purpose of open access, the authors have applied a Creative Commons Attribution (CC BY) license to any author-accepted manuscript version arising from this submission.

***Data accessibility.*** The EPIONCHO-IBM model code is available at: https://github.com/mrc-ide/EPIONCHO.IBM . All information used for the analyses is contained in the figures, tables, and Supplementary Material.

***Copyright.*** 2024 World Health Organization.

This is an open access article distributed under the Creative Commons Attribution IGO License, which permits unrestricted use, distribution, and reproduction in any medium, provided the original work is properly cited. https://creativecommons.org/licenses/by/3.0/igo/ . In any use of this article, there should be no suggestion that the World Health Organization (WHO) endorses any specific organization, products, or services. The use of the WHO logo is not permitted. This notice should be preserved along with the article's original URL.
