# Supplementary material for: An Updated Economic Assessment of Moxidectin Treatment Strategies for Onchocerciasis Elimination
Source: Clin Infect Dis. 2024 Apr 25;78(Suppl 2):S138–45. doi: 10.1093/cid/ciae054 (PMC11045023; doi:10.1093/cid/ciae054)
Supplement: ciae054_Supplementary_Data [file ciae054_supplementary_data.pdf]

Supplementary Material

An updated economic assessment of moxidectin treatment strategies for onchocerciasis elimination

Hugo C. Turner[1,2], Klodeta Kura[1,2], Barbara Roth[3], Annette C. Kuesel[4], Sally Kinrade[3], and Maria-Gloria Basáñez[1,2,*]

[1] MRC Centre for Global Infectious Disease Analysis, Department of Infectious Disease Epidemiology, School of Public Health, Imperial College London, London, UK

[2] London Centre for Neglected Tropical Disease Research, Department of Infectious Disease Epidemiology, School of Public Health, Imperial College London, UK

[3] Medicines Development for Global Health (MDGH), Melbourne, Australia

[4] UNICEF/UNDP/World Bank/WHO Special Programme for Research and Training in Tropical Diseases (WHO/TDR), World Health Organization, Geneva, Switzerland (retired)

* Corresponding author

**Correspondence to:**	**Alternative contact:**
Prof María-Gloria Basáñez	Dr Hugo C. Turner
MRC Centre for Global Infectious Disease Analysis; London Centre for NTD Research<br>Department of Infectious Disease Epidemiology. School of Public Health<br>Imperial College London<br>St Mary's campus<br>Norfolk Place<br>London, W2 1PG, UK	MRC Centre for Global Infectious Disease Analysis; London Centre for NTD Research<br>Department of Infectious Disease Epidemiology. School of Public Health<br>Imperial College London<br>St Mary's campus<br>Norfolk Place<br>London, W2 1PG, UK
Office: +44 20 7594 3295	Office: +44 20 7594 7120
m.basanez@imperial.ac.uk	hugo.turner@imperial.ac.uk

Text S1. Calculation of elimination probabilities and EoT_{90}

The stochastic EPIONCHO-IBM onchocerciasis transmission model [1] was used by Kura et al. [2] to project the number of treatment rounds necessary to achieve of elimination of transmission (EoT) of *Onchocerca volvulus* for a range of strategies and epidemiological scenarios (see Table 1 of Main Text) in endemic (closed) populations of size 440 individuals. Given the stochastic nature of infection events in a model such as EPIONCHO-IBM (with demographic and transmission stochasticity), it is possible for microfilarial prevalence values to fluctuate after cessation of treatment even after the transmission breakpoint has been crossed. Therefore, EoT was considered to have been achieved within a model run when skin microfilarial prevalence was 0%, 50 years after the last MDA treatment round. As the model is stochastic, the number of rounds needed to achieve EOT will vary across different model runs, and the probability of elimination is thus calculated as the proportion of (in 500) runs that achieve EOT as defined above. The probabilities of elimination were calculated after every two years of treatment and plotted against the number of years of mass drug administration (MDA) with ivermectin or moxidectin. For the analysis presented in the Main Text, the number of rounds reported for each scenario was based on the number needed to reach a 90% probability of elimination (EoT_{90}) over such 500 model runs. A detailed description of the methodology for the simulations conducted to investigate the epidemiological impact of moxidectin in comparison to ivermectin MDA can be found in Kura et al. [2].

Text S2. Drug effects

The drug effects of ivermectin had been parameterised by fitting appropriate functions of time following treatment to data obtained from a previous systematic review and meta-analysis of single-dose ivermectin trials (Figure S1A) [3]. Moxidectin was assumed to exert the same types of effects on the parasite as ivermectin, and these effects were parameterised by fitting the same functions as used in Basáñez et al. [3] to the Phase II moxidectin trial data [4] (Figure S1B). As reflected in the error bars presented in Figure S1, there is a greater amount of inter-individual variation in treatment responses to single-dose ivermectin than to single-dose moxidectin (see also Opoku et al. [5]). However, due to the fact that it is still unknown whether the patterns of inter-individual variation observed in the Phase II

and III clinical trials will apply to consecutive treatment rounds, the consequences of such variation in the microfilaricidal and/or embryostatic drug effects was not considered within this study (but see [6]).

The model also incorporates a permanent sterilizing effect of female worms. In the previous modelling study and economic analysis of moxidectin, this effect was referred to as an anti-macrofilarial action of treatment [7]. This action assumes, following Plaisier et al. [8], that repeated exposure of adult worms to treatment permanently reduces their rate of microfilarial production. This reduction was assumed to be 35% per standard (150 µg/kg) dose of ivermectin [8]. As no data yet exist on the effect of multiple doses of (8 mg) moxidectin, we assumed the same magnitude of permanent sterilizing effect as for ivermectin. Given that moxidectin has a longer half-life [9,10], and more prolonged effect on microfilaridermia after a single dose [4,5,11], it is plausible to hypothesize that the permanent sterilizing effect of moxidectin may be greater than that of ivermectin. Although currently there are no data with which to scrutinise this conjecture, we investigated the repercussions of varying the magnitude of the putative permanent sterilizing effect between the two drugs. We considered a scenario in which ivermectin had half the permanent sterilizing effect compared to moxidectin (with ivermectin causing an irreversible reduction in microfilarial production of 17.5% per treatment dose/round, and moxidectin causing 35% reduction per dose/round). Table S1 presents the parameter estimations for the drug effects of ivermectin and moxidectin. Given the uncertainty surrounding the magnitude of this irreversible sterilizing drug effect, the previous economic evaluation of moxidectin conducted by Turner et al. [7] investigated a range of values, from 1% to 30%, with a nominal value of 7% cumulative reduction of microfilarial production, per treatment dose/round, for both ivermectin and moxidectin (see Text S3: Comparison with the previous economic analysis).

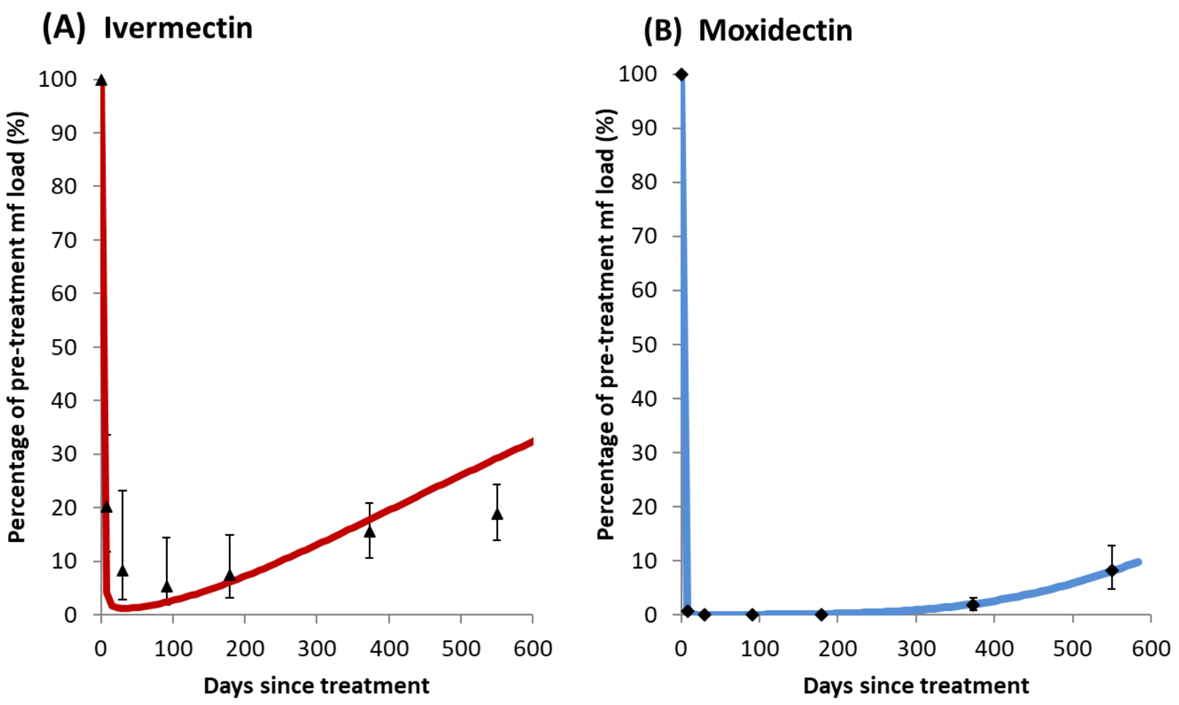

Figure S1. The dynamic effect of a single dose of ivermectin (A) and moxidectin (B) on skin microfilarial load. The data points represent the proportion (in percent) relative to pre-treatment of skin microfilarial loads (the mean of four microfilarial counts/mg, from four (weighed) skin snips per individual participant) collected at 1, 2, 3, 6, 12 and 18 months after treatment from **(A)** the 45 control participants who took a single standard dose, 150 µg/kg of ivermectin) and **(B)** the 38 treated participants who took a single dose, 8mg of moxidectin) as part of the Phase II clinical trial of moxidectin for the treatment of onchocerciasis [4]. The temporal dynamics of a single standard dose of ivermectin, previously parameterized by fitting to microfilarial load data collated as part of a systematic review and modelling meta-analysis [3], is shown as the solid red line in **(A)** by way of validation (i.e., not fitted to the Phase II trial data). The temporal dynamics of a single dose of moxidectin was fitted to the trial data on microfilarial loads from treated participants, and drug effect parameters were estimated [7] using the same approach described in [3], shown as the solid blue line in **(B)**. Error bars are the 95% confidence intervals (95% CIs) around the data at each time-point following treatment. As the variance of microfilarial load for moxidectin is substantially smaller than for ivermectin, the 95% CIs for the moxidectin data points are narrower and may not be visible.

Table S1. Parameter definitions for anti-parasitic effects of ivermectin and moxidectin implemented in EPIONCHO-IBM. Adapted from [2]

Parameter or Variable	Definition	Value, Units and References
$\mu'_M(\tau_{(i)}) = (\tau_{(i)} + \nu)^{-\varpi}$	*Microfilaricidal effect:* Drug-induced per capita rate of excess mortality of *Onchocerca volvulus* microfilariae, μ'_M, at time $\tau_{(i)}$ since treatment	Controlled by ν and ϖ, yr^{-1}
ν	Constant to allow for very large yet finite microfilaricidal effect upon treatment with drug	Ivermectin: 0.0096 [3]<br>Moxidectin: 0.04 [7]
ϖ	Shape parameter for excess mortality of microfilariae following treatment with drug	Ivermectin: 1.25 [3]<br>Moxidectin: 1.82 [7]
$\lambda'_M(\tau_{(i)}) = \lambda^{max} e^{(-\varphi\ \tau(i))}$	*Embryostatic effect:* Drug-induced per capita rate of temporary reversion from fertile (producing microfilariae) to non-fertile adult female worms, λ'_M, at time $\tau_{(i)}$, since treatment	Controlled by λ^{max} and φ, yr^{-1}
λ^{max}	Maximum rate of drug-induced female worm (transient) embryostasis	Ivermectin: 32.4 yr^{-1} [3]<br>Moxidectin: 462 yr^{-1} [7]
φ	Rate of decay of drug-induced female worm (transient) embryostasis	Ivermectin: 19.6 yr^{-1} [3]<br>Moxidectin: 4.83 yr^{-1} [7]
λ'_p	*Permanent sterilizing effect:* Proportion of adult female worms made irreversibly infertile at each treatment round	Ivermectin: 0.345 [8] (or 0.175 in Fig S2A, Table S6A and Table S7A)<br>Moxidectin: 0.345 (assumed to be equal to ivermectin's) as estimated in [8]

Modelling for policy: PRIME-NTD

For the analyses presented, we adhered to the Five Principles of the Neglected Tropical Disease (NTD) Modelling Consortium for good practice in policy-relevant NTD modelling [12]. Table S2 briefly describes the five tenets, how they were fulfilled, and where in the Main Text and/or Supplementary Material they can be found.

Table S2. Policy-Relevant Items for Reporting Models in Epidemiology of Neglected Tropical Diseases (PRIME-NTD) summary table. Following [12]

Principle	What has been done to satisfy the principle?	Where in the manuscript is this described?
**Stakeholder engagement**	Various meetings with members of Medicines Development for Global Health; discussions with WHO/TDR	Author list, Acknowledgements and Funding sections
**Complete model documentation**	References to the full description of the EPIONCHO-IBM model are provided. A link for Open Access to the code has been provided	Methods section, Supplementary Material and Data accessibility section
**Complete description of data used**	Parameters used are described in the manuscript, or pertinent references are cited (Main text and Supplementary Material)	Main text, Supplementary Material, Reference lists
**Communicating uncertainty**	Sensitivity analyses were conducted to investigate the influence on projected costs of varying the increase of yearly cost of biannual treatment and the magnitude of the permanent sterilizing effect of ivermectin	Methods and Results sections. Supplementary Material
**Testable model outcomes**	Model outcomes can be tested when moxidectin is included in treatment guidelines for onchocerciasis elimination and deployed in endemic areas. Projected costs require additional information on the cost of moxidectin to national programmes and the cost of biannual delivery	Discussion and Conclusions sections

Table S3. Projected number of treatments per 100,000 individuals needed to achieve EoT_{90} or reach the 40-year time horizon without achieving EoT_{90} for the endemicity levels, treatment strategies and coverage scenarios investigated

	**(A)** Minimal coverage scenario			**(B)** Enhanced coverage scenario		
Strategy	Baseline prevalence			Baseline prevalence		
	30%	50%	70%	30%	50%	70%
aCDTI	975,000	2,145,000	2,600,000*	960,000	1,680,000	3,200,000*
bCDTI	1,040,000	1,950,000	5,070,000	1,120,000	1,920,000	3,520,000
aCDTM	780,000	1,300,000	2,600,000*	800,000	1,200,000	2,400,000
bCDTM	1,040,000	1,560,000	2,600,000	960,000	1,600,000	2,560,000

(A) Minimal coverage scenario: 65% therapeutic coverage of total population and 5% systematic non-adherence.

(B) Enhanced coverage scenario: 80% therapeutic coverage of total population and 1% systematic non-adherence.

It was assumed that both drugs exert a cumulative, permanent reduction of the rate of microfilarial production by adult female worms of 35% per treatment round with a standard dose of ivermectin (150 µg/kg) or moxidectin (8 mg).

For biannual treatment strategies, results are based on rounding up to whole numbers regarding the number of years of treatment needed to achieve 90% probability of reaching elimination of transmission (EoT_{90}).

* EoT_{90} not attained within the 40-year time horizon.

Table S4. Total (absolute) programmatic delivery cost (in US$) for the different endemicity levels, treatment strategies and coverage scenarios investigated assuming that the total yearly cost would increase by 60% when treating biannually

	**(A)** Minimal coverage scenario		
Strategy	Baseline microfilarial prevalence		
	30%	50%	70%
aCDTI	US$621,382	US$1,080,881	US$1,203,148
bCDTI	US$584,612	US$994,211	US$1,899,506
aCDTM	US$518,116	US$774,388	US$1,203,148
bCDTM	US$584,612	US$828,986	US$1,239,021

	**(B)** Enhanced coverage scenario		
Strategy	Baseline microfilarial prevalence		
	30%	50%	70%
aCDTI	US$518,116	US$802,368	US$1,203,148
bCDTI	US$518,868	US$828,986	US$1,327,253
aCDTM	US$444,006	US$621,382	US$1,020,224
bCDTM	US$451,153	US$710,410	US$1,046,110

(A) Minimal coverage scenario: 65% therapeutic coverage of total population and 5% systematic non-adherence.

(B) Enhanced coverage scenario: 80% therapeutic coverage of total population and 1% systematic non-adherence.

The values represent the projected total programmatic delivery cost of the different treatment strategies over a 40-year time horizon and exclude the economic value of the drugs.

The annual economic cost of the aCDTI strategy was assumed to be US$50,535 per 100,000 individuals.

It was assumed that both drugs exert a cumulative, permanent reduction of the rate of microfilarial production by adult female worms of 35% per treatment round with a standard dose of ivermectin (150 µg/kg) or moxidectin (8 mg).

Values highlighted in green shading indicate scenarios where 90% probability of achieving EoT (EoT_{90}) was attained within 40 years of treatment; values highlighted in pink shading indicate those scenarios for which EoT_{90} was not achieved within 40 years.

Table S5. Total (absolute) programmatic delivery cost (in US$) for the different endemicity levels, treatment strategies and coverage scenarios investigated assuming that the total yearly cost would increase by 100% when treating biannually

	**(A)** Minimal coverage scenario		
Strategy	Baseline microfilarial prevalence		
	30%	50%	70%
aCDTI	US$621,382	US$1,080,881	US$1,203,148
bCDTI	US$730,765	US$1,242,764	US$2,374,383
aCDTM	US$518,116	US$774,388	US$1,203,148
bCDTM	US$730,765	US$1,036,233	US$1,548,776

	**(B)** Enhanced coverage scenario		
Strategy	Baseline microfilarial prevalence		
	30%	50%	70%
aCDTI	US$518,116	US$802,368	US$1,203,148
bCDTI	US$648,586	US$1,036,233	US$1,659,066
aCDTM	US$444,006	US$621,382	US$1,020,224
bCDTM	US$563,941	US$888,012	US$1,307,637

(A) Minimal coverage scenario: 65% therapeutic coverage of total population and 5% systematic non-adherence.

(B) Enhanced coverage scenario: 80% therapeutic coverage of total population and 1% systematic non-adherence.

The values represent the projected total programmatic delivery cost of the different treatment strategies over a 40-year time horizon and exclude the economic value of the drugs.

The annual economic cost of the aCDTI strategy was assumed to be US$50,535 per 100,000 individuals.

It was assumed that both drugs exert a cumulative, permanent reduction of the rate of microfilarial production by adult female worms of 35% per treatment round with a standard dose of ivermectin (150 µg/kg) or moxidectin (8 mg).

Values highlighted in green shading indicate scenarios where 90% probability of achieving EoT (EoT_{90}) was attained within 40 years of treatment; values highlighted in pink shading indicate those scenarios for which EoT_{90} was not achieved within 40 years.

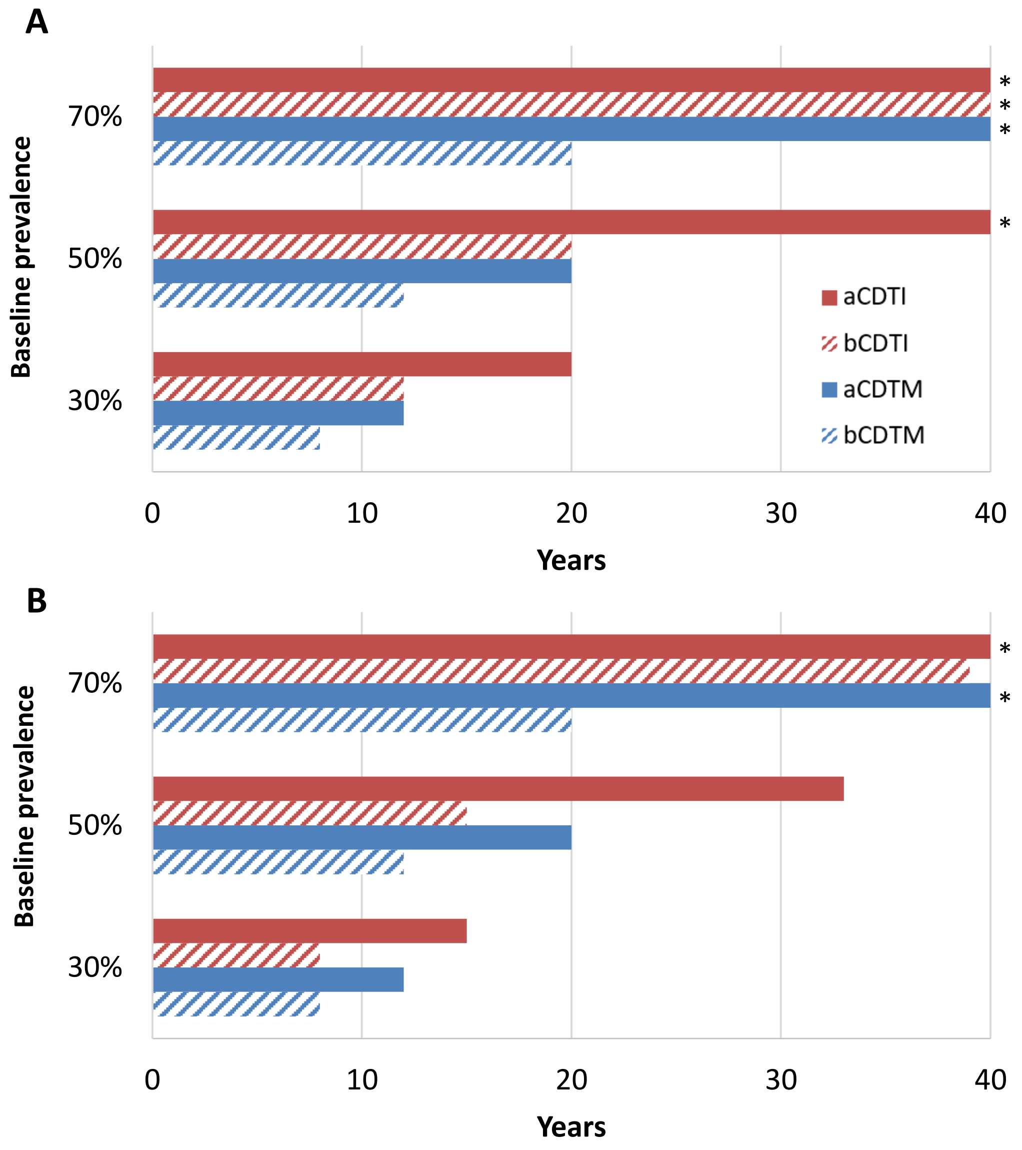

Figure S2. Projected number of years to achieve 90% probability of elimination of transmission (EoT_{90}) for three endemicity levels (baseline microfilarial prevalence) under two assumptions regarding the magnitude of the permanent sterilizing effect on *O. volvulus* of ivermectin and moxidectin assuming minimal coverage. **(A)** A dose (150 µg/kg) of ivermectin irreversibly reduces microfilarial productivity of female worms by 17.5%, whereas a dose (8mg) of moxidectin exerts a reduction of 35%, per treatment round. (**B)** Both ivermectin and moxidectin exert a permanent sterilizing effect of 35% per treatment round. * Elimination not attained within the 40-year time horizon. Minimal coverage: 65% therapeutic coverage of total population and 5% systematic non-adherence.

Table S6. Projected number of years to achieve 90% probability of elimination of transmission (EoT_{90}) and relative programmatic delivery cost of the different treatment strategies compared to aCDTI over the 40-year time-horizon, under minimal coverage scenario, when varying the magnitude of the permanent sterilizing effect on *O. volvulus* adult female worms and assuming that biannual delivery increases the yearly cost of treatment by 60%

(A) Treatment duration (in years) and relative cost (in percent) compared to aCDTI assuming that ivermectin has half the magnitude of the permanent sterilizing effect compared to moxidectin

Strategy	Baseline prevalence		
	30%	50%	70%
aCDTI	20 (0%)	40+ (0%)	40+ (0%)
bCDTI	12 (+7%)	20 (+3%)*	40+ (+60%)*
aCDTM	12 (–33%)	20 (–36%)*	40+ (0%)*
bCDTM	8 (–25%)	12 (–31%)*	20 (+3%)*

(B) Treatment duration (in years) and relative cost (in percent) compared to aCDTI assuming that ivermectin and moxidectin have the same magnitude of the permanent sterilizing effect

Strategy	Baseline prevalence		
	30%	50%	70%
aCDTI	15 (0%)	33 (0%)	40+ (0%)
bCDTI	8 (–6%)	15 (–8%)	39 (+58%)*
aCDTM	12 (–17%)	20 (–28%)	40+ (0%)*
bCDTM	8 (–6%)	12 (–23%)	20 (+3%)*

The values in brackets represent the projected relative proportions (in percent) of total programmatic delivery cost of the different treatment strategies compared to aCDTI over a 40-year time horizon and exclude the economic value of the drugs. Values of 0% signify that the costs are the same as aCDTI; those accompanied by a minus sign indicate a relative reduction in cost; those accompanied by a plus sign indicate a relative increase in cost.

(A) The cumulative, permanent reduction in microfilarial production is 17.5% per treatment round for ivermectin and 35% per round for moxidectin.

(B) The cumulative, permanent reduction in microfilarial production is 35% per treatment round for both drugs.

Minimal coverage scenario: 65% therapeutic coverage of total population and 5% systematic on-adherence.

*As EoT was not attained with aCDTI within the 40-year time horizon, the relative costs are calculated based on costs of 40 years of aCDTI.

The corresponding projections of the total programmatic delivery costs are presented in Table S7.

Table S7. Total (absolute) programmatic delivery cost (in US$) for the different endemicity levels and treatment strategies, under minimal coverage scenarios when varying the magnitude of the permanent sterilizing effect on *O. volvulus* adult female worms and assuming that biannual delivery increases the yearly cost of treatment by 60%

(A) Ivermectin has half the magnitude of the permanent sterilizing effect compared to moxidectin

Strategy	Baseline microfilarial prevalence		
	30%	50%	70%
aCDTI	US$774,388	US$1,203,148	US$1,203,148
bCDTI	US$828,986	US$1,239,021	US$1,925,037
aCDTM	US$518,116	US$774,388	US$1,203,148
bCDTM	US$584,612	US$828,986	US$1,239,021

(B) Ivermectin and moxidectin have the same magnitude of the permanent sterilizing effect

Strategy	Baseline microfilarial prevalence		
	30%	50%	70%
aCDTI	US$621,382	US$1,080,881	US$1,203,148
bCDTI	US$584,612	US$994,211	US$1,899,506
aCDTM	US$518,116	US$774,388	US$1,203,148
bCDTM	US$584,612	US$828,986	US$1,239,021

The values represent the projected total programmatic delivery cost of the different treatment strategies over a 40-year time horizon and exclude the economic value of the drugs.

(A) The cumulative, permanent reduction in microfilarial production is 17.5% per treatment round for ivermectin and 35% per round for moxidectin.

(B) The cumulative, permanent reduction in microfilarial production is 35% per treatment round for both drugs.

Minimal coverage scenario: 65% therapeutic coverage of total population and 5% systematic on-adherence.

Values highlighted in green shading indicate scenarios where 90% probability of achieving EoT (EoT_{90}) was attained within 40 years of treatment; values highlighted in pink shading indicate those scenarios for which EoT90 was not achieved within 40 years.

Text S3. Comparison with the previous economic analysis

The modelling by Turner et al. [7] projected that aCDTM and bCDTI would achieve similar reductions in programme duration relative to aCDTI, which was reflected in the estimated relative (to aCDTI) delivery costs. Within this updated analysis, the results pertaining to the benefit of aCDTM relative to bCDTI differ to a greater extent. This is due to two key differences: 1) the previous modelling analysis used a version of the population-based, deterministic EPIONCHO model, that by its nature, only enabled determination of the number of treatment rounds needed to achieve microfilarial prevalence thresholds in the context of the provisional Operational Thresholds for Treatment Interruption and commencement of Surveillance (pOTTIS) proposed by the African Programme for Onchocerciasis control (APOC) [13]. By contrast, this analysis used the individual-based, stochastic onchocerciasis transmission EPIONCHO-IBM model [1], which projected the number of treatment rounds needed to reach 90% probability of EoT (EoT_{90}) [2]; 2) the base case results in the previous modelling analysis assumed a lower permanent sterilizing effect (a 7% cumulative reduction in microfilarial production per treatment round/dose for both ivermectin and moxidectin, compared to the 35% assumed in this analysis). Thishigher, assumed magnitude of the permanent sterilizing effect, increases the modelled impact of bCDTI.

Notwithstanding these differences, the results presented here support the notion that moxidectin MDA treatment could accelerate progress towards onchocerciasis EoT and generate savings in programmatic delivery costs based on the assumptions used.

Supplementary References

1. Hamley JID, Milton P, Walker M, Basáñez MG. Modelling exposure heterogeneity and density dependence in onchocerciasis using a novel individual-based transmission model, EPIONCHO-IBM: implications for elimination and data needs. PLoS Negl Trop Dis **2019;** 13: e0007557.

2. Kura K, Milton P, Hamley JID et al. Can mass drug administration of moxidectin accelerate onchocerciasis elimination in Africa? Philos Trans R Soc Lond B Biol Sci **2023;** 378: 20220277.

3. Basáñez MG, Pion SDS, Boakes E, Filipe JAN, Churcher TS, Boussinesq M. Effect of single-dose ivermectin on *Onchocerca volvulus*: a systematic review and meta-analysis. Lancet Infect Dis **2008;** 8: 310–22.

4. Awadzi K, Opoku NO, Attah SK, Lazdins-Helds J, Kuesel AC. A randomized, single-ascending-dose, ivermectin-controlled, double-blind study of moxidectin in *Onchocerca volvulus* infection. PLoS Negl Trop Dis **2014;** 8: e2953.

5. Opoku NO, Bakajika DK, Kanza EM, et al. Single dose moxidectin versus ivermectin for *Onchocerca volvulus* infection in Ghana, Liberia, and the Democratic Republic of the Congo: a randomised, controlled, double-blind phase 3 trial. Lancet **2018;** 392: 1207–16.

6. Milton P. Mathematical modelling to support the elimination of onchocerciasis transmission. PhD thesis. Imperial College London, **2022**.

7. Turner HC, Walker M, Attah SK, et al. The potential impact of moxidectin on onchocerciasis elimination in Africa: an economic evaluation based on the Phase II clinical trial data. Parasit Vectors **2015;** 8:167.

8. Plaisier AP, Alley ES, Boatin BA, et al. Irreversible effects of ivermectin on adult parasites in onchocerciasis patients in the Onchocerciasis Control Programme in West Africa. J Infect Dis **1995;** 172:204–10.

9. Milton P, Hamley JID, Walker M, Basáñez MG. Moxidectin: an oral treatment for human onchocerciasis. Expert Rev Anti Infect Ther **2020;** 18:1067–81.

10. Tan B, Opoku N, Attah SK, et al. Pharmacokinetics of oral moxidectin in individuals with *Onchocerca volvulus* infection. PLoS Negl Trop Dis **2022;** 16: e0010005.

11. Bakajika D, Kanza EM, Opoku NO, et al. Effect of a single dose of 8 mg moxidectin or 150 µg/kg ivermectin on *O. volvulus* skin microfilariae in a randomized trial: differences between areas in the Democratic Republic of the Congo, Liberia and Ghana and impact of intensity of infection. PLoS Negl Trop Dis. **2022;** 16: e0010079.

12. Behrend MR, Basáñez MG, Hamley JID, et al. Modelling for policy: the five principles of the Neglected Tropical Diseases Modelling Consortium. PLoS Negl Trop Dis **2020;** 14: e0008033.

13. World Health Organization & African Programme for Onchocerciasis Control. Conceptual and operational framework of onchocerciasis elimination with ivermectin treatment. African Programme for Onchocerciasis Control. 2010. Available at: https://apps.who.int/iris/handle/10665/275466. Accessed 18 January 2024.
